# Health, Safety, and Wellness Concerns Among Law Enforcement Officers: An Inductive Approach

**DOI:** 10.1177/21650799221134422

**Published:** 2022-12-14

**Authors:** Sébastien Poirier, Noémie Allard-Gaudreau, Philippe Gendron, Julie Houle, François Trudeau

**Affiliations:** 1 Université du Québec en Abitibi-Témiscamingue; 2 Université du Québec à Trois-Rivières

**Keywords:** law enforcement officers, health, safety, and wellness, content analysis, workplace health and safety

## Abstract

*Background*: Although studies have assessed the impact of occupational risk factors on the health of law enforcement officers (LEO’s), few have involved (LEO’s) as informants in ways that allow their points of view to be heard directly. Thus, the objective of this study is to explore the occupational health, safety, and wellness (OHSW) concerns of (LEO’s). *Methods*: (LEO’s) working in Quebec, Canada were invited to answer an open-ended question regarding their OHSW concerns. Using a multi-stage content analysis, the collected answers were analyzed and coded by two members of the research team to identify the most recurrent concerns of (LEO’s). *Findings*: Five themes relating to the OHSW concerns of (LEO’s) were identified, namely, the *work schedule*, *occupational stress*, *work equipment*, *workplace health promotion*, and *operational risks*. Furthermore, our analyses highlighted differences in the concerns of (LEO’s) based on their level of experience and sex. *Conclusions/Application to Practice*: This study addresses a gap in the literature on the OHSW concerns from the perspective of (LEO’s). Overall, our results support that the work schedule and occupational stress associated with law enforcement are the two most recurrent concerns of (LEO’s). Thus, the results of this study further stress the need for police organizations to implement strategies and policies, which could mitigate the deleterious effects of these hazards on the overall wellness of (LEO’s).

## Background

The overall rate of nonfatal work injuries is three times higher in U.S law enforcement officers (LEO’s) compared with other workers (Tiesman et al., 2018). Furthermore, statistics reported by the National Law Enforcement Officers Memorial Fund show that, in 2020, 295 LEO’s died while on duty in the United States (National Law Enforcement Officers Memorial Fund, 2021). In addition to these operational risks, other detrimental health outcomes including cardiovascular diseases and related risk factors have been associated with law enforcement (Gendron et al., 2019; Lee et al., 2022; Rostami et al., 2019; Violanti, 2014). Previous studies also report that (LEO’s) suffer from chronic pain, including back pain (Benyamina Douma et al., 2017; Carleton et al., 2017) and have a higher incidence of mental health problems compared with the general population (MacEachern et al., 2019).

Worksite wellness programs have been shown to have beneficial impacts on the mental and physical health of (LEO’s) (Acquadro Maran et al., 2018; Antony et al., 2020; MacMillan et al., 2017; Taylor et al., 2021). Nevertheless, most law enforcement agencies do not currently offer wellness programs (Taylor et al., 2021). Although previous studies have assessed the impact of some occupation-specific risk factors on the health of LEO’s, few have involved LEO’s as participants in ways that allow their point of view to be heard directly. The Total Worker Health^®^ framework (Lee et al., 2016) suggests that LEO’s perceptions of their own occupational health, safety, and wellness (OHSW) needs could inform the development of OHSW programs by providing a holistic understanding of the factors associated with their well-being. Furthermore, involving workers throughout the development of wellness programs is believed to nurture workers’ engagement in future programs and increase their long-term effectiveness (Feltner et al., 2016; Lee et al., 2016). The purpose of this inductive study is to explore the OHSW concerns of LEO’s in Québec.

## Methods

### Study Design and Measures

This descriptive study utilized the inductive analysis of a dataset previously collected during survey-based research on the health and wellness of LEO’s (Gendron et al., 2019). During this previous research, several questions were used to assess the health status and working conditions of LEO’s. To explore the OHSW concerns of participants, one open-ended question was included at the end of the questionnaire (Would you like to share any concerns or points of view on the OHSW of police officers in Québec? If so, we would gladly consider them.). This study utilizes both qualitative and quantitative analyses to assess the narrative responses to the open-ended question.

**Applying Research to Occupational Health Practice**

Our results show that the most recurrent occupational health, safety, and wellness (OHSW) concerns of law enforcement officers (LEO’s) were *the work schedule*, *occupational stress*, *work equipment*, *workplace health promotion*, and *operational risks*. Our analyses also highlighted some variations in the concerns of LEO’s based on their level of experience and biological sex. As concerns relating to the work schedule were by far the most recurrent, organizations should strongly consider the implementation of interventions to minimize the adverse effects of shift work such as fatigue-management education trainings, flexible shift arrangements and positive coping strategies. Furthermore, while the literature on police-related stress has mostly focused on the operational stress experienced by LEO’s, our results highlight the stress-inducing potential of police organizational structure and management practices. Thus, the implementation of strategies to eliminate or reduce sources of organizational stress should be prioritized in police organizations.

### Sample

The entire police population in Québec (*N* = 15,159) was invited to complete an online questionnaire through their police organizations. This study was approved by the Human Research Ethics Committee of the Université du Québec à Trois-Rivières. All participants provided written informed consent prior to the study.

#### Qualitative Analysis

Inductive content analysis was used to analyze the qualitative data. Content analysis is a systematic coding and categorizing approach used to determine trends and patterns of words in textual information (Vaismoradi et al., 2013). All answers were independently read by two members of our research team to identify recurrent and salient OHSW concerns. To do so, in vivo coding was used, meaning that codes were created during the analytic process based on the actual words of participants (Saldaña, 2011). Following this first coding phase, the various and numerous codes created were discussed by team members. From a mutual agreement, similar codes were combined into five overarching codes or “categories.” All answers were then once again read and classified into one or more of these five categories. Although most participants identified only one concern, answers comprising more than one statement relating to different concerns were classified into multiple categories. The content analysis was performed using Microsoft Excel (2016). It should be noted that both the open-ended question and the answers provided by our participants were in French language.

#### Quantitative Analyses

Following the content analysis, the frequency of occurrence of each category was computed. Although the importance of the different categories is not solely dependent on their prevalence, such quantifiable measures can be useful to demonstrate how widespread themes or patterns of meaning are across a data set (Vaismoradi et al., 2013). During the coding process, it appeared to team members that the identified OHSW concerns differed based on the biological sex and the level of experience of participants. Thus, statistical analyses were performed to investigate the relation of biological sex and length-of-service with the concerns addressed by the participants. First, the proportion of male and female participants who discussed each category of concerns was compared using chi-square analyses. Effect sizes for chi-square analyses were assessed based on Cramer’s *V* (*V*) and interpreted according to Cohen (1988) as small (*V* = 0.10), medium (*V* = 0.30), and large (*V* = 0.50). To assess the relation of length-of-service with the concerns identified, three length-of-service groups were created (<10 years, 10–20 years, and >20 years). The proportion of participants who discussed each category of concerns was once again compared across the three groups using chi-square analyses. All statistical analyses were computed using the Statistics Package for Social Sciences (SPSS Version 27.0; IBM Corporation, New York, NY, USA).

## Results

A total of 2,099 male officers and 756 female officers completed the questionnaire for a total sample size of 2,855 (18.8%). Among them, 404 LEO’s (14.2%) provided an answer to our open-ended question. The characteristics of participants who answered the open-ended question along with those who participated in the previous study but did not answer our question are shown in Table 1.

**Table 1.** Comparison of Participants Who Answered and Did Not Answer the Open-Ended Question

Variables	Total (*n* = 2,855)	Did not answer (*n* = 2,451)	Answered (*n* = 404)	*p* value*
Age (years)	40.0 ± 8.9	40.2 ± 8.9	39.3 ± 8.8	.08
Length-of-service (years)	16.6 ± 8.6	16.7 ± 8.6	15.7 ± 8.4	.03
Biological sex				.02
Male	2,099 (73.5%)	1,782 (72.7%)	317 (78.5%)	
Female	756 (26.5%)	669 (27.3%)	87 (21.5%)	

*Note.* *P values are from independent-samples *t* test (age and length-of-service) or chi-square test (sex).

Based on the content analysis, five salient categories were identified by our research team: *work schedule*, *occupational stress*, *work equipment*, *workplace health promotion*, and *operational risks.* Although some participants merely identified different characteristics of police work that they consider detrimental to their health, safety, or wellness, most participants provided detailed information on how these characteristics affected their life. Thus, as shown in Table 2, our team was able to identify subcategories commonly associated with each main category. The categories and subcategories are further described in the next sections.

**Table 2.** Occupational Health, Safety, and Wellness Concerns: Chi-Square Comparison Between Females and Males

Categories and subcategories	Total (*n* = 404)	Females (*n* = 87)	Males (*n* = 317)	χ^2^	*p* value
Work schedule	191 (47.3)	42 (48.3)	149 (47.0)	0.044	.83
Work–life balance	45 (11.1)	16 (18.4)	29 (9.1)	5.892	.01
Inability to maintain a healthy lifestyle	20 (5.0)	6 (6.9)	14 (4.4)	0.892	.35
Inability to recover between shifts	57 (14.0)	13 (14.9)	44 (13.9)	0.064	.80
Physical and mental health	67 (16.6)	15 (17.2)	52 (16.4)	0.035	.85
Occupational stress	115 (28.5)	27 (31.0)	88 (27.8)	0.359	.55
Operational stress	36 (8.9)	9 (10.3)	27 (8.5)	0.281	.60
Organizational stress	58 (14.4)	13 (14.9)	45 (14.2)	0.031	.86
Public perception	30 (7.4)	5 (5.7)	25 (7.9)	0.454	.50
Work equipment	58 (14.4)	16 (18.4)	42 (13.2)	1.468	.23
Ergonomic factors	54 (13.4)	15 (17.2)	39 (12.3)	1.438	.23
Workplace health promotion	57 (14.1)	7 (8.0)	50 (15.8)	3.363	.07
Enabling factors for physical activity	32 (7.9)	4 (4.6)	28 (8.8)	1.679	.20
Reinforcing factors for physical fitness	21 (5.2)	3 (3.4)	18 (5.7)	0.689	.41
Operational risks	29 (7.2)	7 (8.0)	22 (6.9)	0.125	.72
Distribution of the workforce	11 (2.7)	2 (2.3)	9 (2.8)	0.075	.78
Sudden physical efforts	8 (2.0)	0 (0.0)	8 (2.5)	2.240	.13

*Note.* Results reported as *n* (%).

### Work Schedule

The *work schedule* was the most recurrent category of concerns identified during our analysis. Indeed, 191 of the 404 participants (47.3%) identified the *work schedule* as an OHSW concern. Based on chi-square analyses, the proportion of participants who discussed the *work schedule* was similar for male and female participants but varied across the length-of-service groups (χ^2^ = 14.928, *df* = 2, *p* < .01, *V* = 0.19) with a smaller proportion of LEO’s with more than 20 years of experience discussing this category of concerns (Table 3).

**Table 3.** Occupational Health, Safety, and Wellness Concerns: Chi-Square Comparison Across Length-of-Service Groups

Categories and subcategories	<10 years (*n* = 133)	10–20 years (*n* = 142)	>20 years (*n* = 129)	χ^2^	*p* value
Work schedule	70 (52.6)	78 (54.9)	43 (33.3)	14.928	<.01
Work–life balance	19 (14.3)	20 (14.1)	6 (4.7)	8.061	.02
Inability to maintain a healthy lifestyle	11 (8.3)	2 (1.4)	7 (5.4)	6.964	.03
Inability to recover between shifts	23 (17.3)	23 (16.2)	11 (8.5)	4.940	.09
Physical and mental health	28 (21.1)	23 (16.2)	16 (12.4)	3.565	.17
Occupational stress	26 (19.5)	42 (29.6)	47 (36.4)	9.302	.01
Operational stress	4 (3.0)	13 (9.2)	19 (14.7)	11.100	<.01
Organizational stress	14 (10.5)	20 (14.1)	24 (18.6)	3.489	.18
Public perception	11 (8.3)	10 (7.0)	9 (7.0)	0.206	.90
Work equipment	28 (21.1)	17 (12.0)	13 (10.1)	7.428	.02
Ergonomic factors	26 (19.5)	15 (10.6)	13 (10.1)	6.559	.04
Workplace health promotion	16 (12.0)	16 (11.3)	25 (19.4)	4.378	.11
Enabling factors for physical activity	10 (7.5)	11 (7.7)	11 (8.5)	0.100	.95
Reinforcing factors for physical fitness	6 (4.5)	5 (3.5)	10 (7.8)	2.645	.27
Operational risks	13 (9.8)	8 (5.6)	8 (6.2)	2.038	.36
Workforce distribution	3 (2.3)	5 (3.5)	3 (2.3)	0.528	.77
Sudden physical efforts	4 (3.0)	3 (2.1)	1 (0.8)	1.701	.43

*Note.* Results reported as *n* (%).

Four recurrent subcategories were associated with the *work schedule*, namely, the *physical and mental health*, the *inability to recover between shifts*, the *work–life balance*, and the *inability to maintain a healthy lifestyle. Physical and mental health* was the subcategory most often associated with the work schedule (*n* = 67, 16.6%). This subcategory included all participants, which directly attributed their poor physical and mental health status to the work schedule:

It is certain that health takes a hit, unless you have a meal plan and a solid physical exercise routine, I believe that a police officer cannot work during his entire career on the three shifts (day, evening, and night), the body simply does not follow after 8-10 years of career on the roads. (Male, 22 years of service)

The *inability to recover between shifts* was another recurrent subcategory associated with the work schedule (*n* = 57, 14.0%). Many participants mentioned their inability to get enough sleep between consecutive night shifts. Others mentioned having developed sleep disorders, mainly difficulty falling and staying asleep, as a result of the rotating schedule. Excessive sleepiness and mood problems were often reported as direct consequences of these sleep disorders:

At this rate, I will never get to 20 years of service. The rotating shifts are killing me, literally. When I am working nights, I am in survival mode. I can’t sleep, period. Then when I’m working daytimes, my body can’t get back to normal. I wake up 4 to 5 times a night. I am continually tired and exhausted. (Male, 6 years of service)

The difficulty to maintain a proper *work–life balance* was also discussed by many participants (*n* = 45, 11.1%). The difficulty to fulfill family responsibilities and maintain a satisfying social life because of the rotating work schedule and the total workload were mainly discussed. Small but significant differences in the proportion of participants who discussed *work–life balance* as a concern associated to the work schedule were found across the length-of-service groups (χ^2^ = 8.061, *df* = 2, *p* = .02, *V* = 0.14). Interestingly, a significantly larger proportion of female LEO’s mentioned that the *work–life balance* was a downfall of their work schedule (χ^2^ = 5.892, *df* = 1, *p* = .01, *V* = 0.12). Female LEO’s mainly mentioned the difficulty to juggle between their parental and work obligations:

Work-family balance is the most negative aspect of work. This is what causes most of my stress and lack of sleep. Without family obligations, night work is easier since no one depends on you . . . (Female, 12 years of service)

The more the years go on, the more difficult it is to work at night. [. . .] I love my job, but having the financial possibility of changing my job, I would. I can feel it in the behavior of my children, they miss their mom. It’s not always easy. (Female, 9 years of service)

Finally, the *inability to maintain a healthy lifestyle* because of the workload and rotating work shifts was also a concern for a considerable proportion of participants (*n* = 20, 5.0%). Participants mostly discussed the difficulty to eat well and stay physically active while working night shifts. The proportion of participants who mentioned the *difficulties to maintain a healthy lifestyle* because of the work schedule was similar for male and female participants but differed based on length-of-service with a smaller proportion of LEO’s with 10 to 20 years of experience bringing up this subcategory (χ^2^ = 6.964, *df* = 2, *p* = .03, *V* = 0.13).

### Occupational Stress

The category *occupational stress* comprised all answers describing a feeling of stress, a state of anxiety, or a negative emotional response relating to law enforcement. A total of 115 participants (28.5%) identified *occupational stress* as a concern for their health, safety, or wellness. Chi-square analyses showed that the proportion of participants who discussed *occupational stress* was similar among both sexes but differed across the different length-of-service groups (χ^2^ = 9.302, *df* = 2, *p* = .01, *V* = 0.15). Indeed, the proportion of less experienced LEO’s (<10 years of service) who mentioned *occupational stress* in their answer was significantly smaller compared with more experienced LEO’s.

Three subcategories for *occupational stress* were identified during our analyses: *organizational stress*, *operational* stress, *and public perception. Organizational stress*, defined as stressors generated by the organization and culture within which LEO’s perform their job, was the most recurrent subcategory (*n* = 58, 14.4%). Among the organizational stressors mentioned, the lack of support and recognition from superiors were often identified as stressors. Furthermore, many LEO’s reported that the presence of productivity “quotas” or “goals” was an important source of stress and frustration:

The negative [aspects] that we are confronted to during our interventions is manageable, it is the negative [aspects] coming from the employer which gnaws at me from the inside. Without any recognition, no support, the work I loved became my cancer. (Male, 27 years of service)

A total of 36 LEO’s (8.9%) identified *operational stress* as a concern for their wellness. This subcategory included all sources of stress inherent to the nature of police tasks and interventions (i.e., exposure to traumatic events). When referring to this subcategory, some LEO’s stated living with symptoms of post-traumatic stress disorder. Meanwhile, the rising complexity of police tasks and the fear of making a mistake during critical interventions were identified as stressful by multiple participants. A larger proportion of LEO’s with more than 20 years of service discussed the *operational stress* compared with less experienced officers (χ^2^ = 11.100, *df* = 2, *p* < .01, *V* = 0.17).

The third subcategory of *occupational stress* identified during our analyses was the *public perception*. The *public perception* was mentioned by 30 participants (7.4%) identifying the unfair media coverage, increased public scrutiny, and overall negative public opinion of law enforcement as important sources of stress affecting their mental health:

The negativism of the population towards police officers affects the morale of police officers. The organization’s lack of recognition and support when negative elements are conveyed in the media discourages and demoralizes many [officers]. (Male, 8 years of service)

### Work Equipment

The *work equipment* was the third most recurrent category identified during our analyses (*n* = 58, 14.4%). Chi-square analyses showed that the proportion of participants who discussed the *work equipment* was similar across males and females but varied across the different length-of-service groups (χ^2^ = 7.428, *df* = 2, *p* = .02, *V* = 0.14). Indeed, less experienced LEO’s (<10 years of experience) seem to attribute greater importance to the *work equipment* as a concern for their health, safety or wellness.

Upon analysis of the answer included in the *work equipment* category, it appeared that nearly all answers discussed *ergonomic factors* associated with the development of chronic pain. Mainly, the size and configuration of the duty belt were identified by many as risk factors for the development of chronic back pain. In this regard, many of the participants suggested that relocating some of the equipment from the duty belt to the protective vest could help reduce discomfort. The patrol car design was also identified by many as contributing to the development of back pain. Mainly, the need to adapt the seats of patrol cars to accommodate for the duty belt and protective vest was discussed by participants.

### Workplace Health Promotion

The fourth category identified during our analyses includes all concerns relating to the lack of *workplace health promotion* among police organizations (*n* = 57, 14.1%). The proportion of participants discussing the *workplace health promotion* was similar among both sexes and across the different levels of experience. Two subcategories were identified, namely, the lack of *enabling factors for physical activity* and *reinforcing factors for physical fitness*. The lack of *enabling factors for physical activity* was the most recurrent subcategory associated with the *workplace health promotion* with 32 participants (7.9%) discussing this concern. Enabling factors are antecedents to the behavior that allow or facilitate a motivation to be realized (Green & Kreuter, 2005). They include any resources and environmental characteristics which enable or facilitate the realization of a specific behavior. Our participants mainly identified two enabling factors which they perceive could help LEO’s stay physically active: providing exercise facilities in police stations and allocating time for LEO’s to exercise while at work:

No possibility to train on premises at the [police organization]. The organization does not want to establish exercise facilities in the stations so that its police officers stay fit. It’s hard to keep a good training routine when we are working on shifts. (Male, 3 years of service)

The second subcategory relating to *workplace health promotion* concerned *reinforcing factors for physical fitness* (*n* = 21, 5.2%). Reinforcing factors are those factors that provide continuing incentive for the persistence or repetition of the behavior (Green & Kreuter, 2005). The answers provided by our participants were mostly focused on the need for fitness assessments to ensure that LEO’s are physically fit-for-duty throughout their entire career. Participants mentioned that physical training should be mandatory for LEO’s found to be unfit to perform police tasks:

We must pass physical tests to go to the police academy. Unfortunately, after your first day at work, nothing forces you to keep your physical condition when it is a requirement at the start. The police departments should at least maintain a physical standard for a certain number of years. Not everyone has the will to do the minimum to stay fit and physically healthy . . . (Female, 27 years of service)

### Operational Risks

The last and least frequent category identified by our research team was the *operational risks* defined as the physical safety concerns associated with the performance of police interventions. A total of 29 participants (7.2%) discussed the operational risks of law enforcement in their answers. The proportion of participants who discussed this category was similar for both sexes and across the different length-of-service groups.

Two subcategories were identified for the *operational risks*: the *distribution of the workforce* and *the sudden physical efforts.* The *workforce distribution* is the subcategory that was the most frequently associated with the *operational risks* (*n* = 11, 2.7%). Overall, two concerns relating to the *distribution of the workforce* were mentioned by our participants. First, some participants mentioned that the frequent single officer patrols expose them to unnecessary safety risks. Thus, the perceived safety of LEO’s seems to be reduced when patrolling alone. Moreover, other participants mentioned that the number of patrol units simultaneously on duty is sometimes insufficient to properly respond to critical incidents. The second subcategory identified was the *sudden physical efforts* (*n* = 8, 2.0%). Indeed, some participants mentioned that the physically demanding interventions often occurring without any warning can expose officers to musculoskeletal injuries.

## Discussion

This article reports the analysis of answers provided by LEO’s to an open-ended question regarding their own OHSW concerns. Using an inductive approach, this qualitative descriptive study allowed the identification of five recurrent categories of concerns, namely, the *work schedule*, *occupational stress*, *work equipment*, *workplace health promotion*, and *operational risks*. Furthermore, our analyses highlighted some variations in the OHSW concerns of LEO’s based on their level of experience and biological sex.

The most frequent concerns mentioned by our participants were related to the work schedule. This is not so surprising given that several studies have highlighted the negative impacts of atypical work schedules on the physical and psychosocial health of workers, including LEO’s (Garbarino et al., 2019; Matheson et al., 2014; Peterson et al., 2019; Torquati et al., 2018). Our analyses showed that a significantly smaller proportion of LEO’s with more than 20 years of experience identified the work schedule as an OHSW concern. This is somewhat expected given that, usually, more experienced LEO’s work night shifts less frequently than younger LEO’s. They are also less likely to have young children, which can exacerbate work–family conflicts (Crompton & Lyonette, 2006).

Perhaps one of the most interesting findings of this study is the sex-based difference found in the subcategories associated with the work schedule. Indeed, although a similar proportion of male and female participants discussed the work schedule, a larger proportion of female LEO’s mentioned that work–life balance was an important OHSW concern associated with their work schedule. Specifically, female LEO’s emphasized the difficulty of simultaneously fulfilling both parental and work responsibilities. This result further highlights the unresolved inequalities in work/family balance with disproportionate load of unpaid work and care generally undertaken by females (Sullivan, 2019). As increased gender diversity in the police forces positively affects police organizations in many ways (Barnes et al., 2018; Brown & Silvestri, 2020; Rabe-Hemp, 2008; Schuck, 2014), these results highlight the need to better understand the unique challenges faced by females in the police workforce.

Occupational stress was another recurrent category identified by our participants. Based on the answers provided by our participants, three subcategories corresponding to different sources of stress were identified, namely, organizational stressors, operational stressors, and public perception. Among these, organizational stressors (stressors generated by the organization and culture within which LEO’s perform their job) were the most frequently mentioned source of stress. This is somewhat consistent with previous results suggesting that, while operational stress is associated with the mental health of LEO’s (Queirós et al., 2020), organizational stress is more closely related to the mental health status of these workers (Baka, 2015; Wolter et al., 2018). The frequency of exposure to the various stressors could very well explain these results. Indeed, previous studies on LEO’s suggest that the level of perceived stress related to particular stressors is strongly correlated with the frequency of exposure to the stressor (McCreary & Thompson, 2006). Thus, although some operational stressors have a strong anxiety-inducing potential, the more frequent exposure to stressors such as perceived pressure from superiors and excessive administrative duties could lead LEO’s to perceive greater levels of stress related to organizational stressors. Moreover, our results show that a larger proportion of experienced LEO’s (≥20 years of experience) discussed the operational stress compared with less experienced officers. Although our analyses cannot account for this difference, given their long career, older LEO’s are more likely to have been exposed to traumatic events and, thus, be dealing with the negative repercussions of these events.

The third category of concerns identified in this study is the work equipment, mostly relating to ergonomic factors associated with the development of chronic pain. Results from multiple epidemiological studies suggest that musculoskeletal pain, specifically low back pain (LBP), is highly prevalent among the police force (Benyamina Douma et al., 2017; Carleton et al., 2017). Although LEO’s are exposed to risk factors associated with LBP such as prolonged driving and psychological stress (Chen et al., 2005; Vinstrup et al., 2020), our results suggest that LEO’s mainly attribute their musculoskeletal pain to the personal protective equipment and patrol car seats. Interestingly, previous results have indicated that lumbar support systems integrated into the backrest of seats (Donnelly et al., 2009; Gruevski et al., 2016; Holmes et al., 2013) and reduced duty belts (Holmes et al., 2013; Larsen et al., 2019) have the potential to reduce self-reported discomfort and lower back pressure while sitting in LEO’s. Thus, as suggested by many of our participants, it appears that modifying the design of patrol cars and personal protective equipment could help reduce the risk of chronic pain in LEO’s.

Workplace health promotion was the fourth category of concerns identified during our analysis. The answers provided by our participants were mainly related to the lack of enabling factors for physical activity and reinforcing factors for physical fitness. Previous results suggest that participation in workplace-based structured physical activity programs can lead to both physical and psychological health improvements in LEO’s and police trainees (Acquadro Maran et al., 2018; Cocke et al., 2016; Gerber et al., 2010; Orr et al., 2016; Rossomanno et al., 2012). Although these results support the beneficial effects of regular physical activity in LEO’s, little is known on the overall impact of these training programs on the short- and long-term physical activity participation of LEO’s. In other words, while there is a considerable amount of literature supporting “why” LEO’s should engage in physical activity, few studies have focused on “how” to engage LEO’s in physical activity. A recent study from Oliver et al. (2022) suggests that an app-based physical activity intervention can increase short-term physical activity participation in LEO’s. Nevertheless, the long-term effects of such intervention are still unknown. In the present study, many participants suggested that providing workplace exercise facilities and allocating time to exercise while at work could facilitate physical activity participation in LEO’s. This is somewhat consistent with the fact that participants identified the work schedule as an important barrier to physical activity participation. Prior study from Gendron et al. (2020) showed that firefighters who exercise while on duty report higher weekly physical activity levels and have better cardiovascular health indicators. However, to our knowledge, no study has yet assessed the impact of providing workplace exercise facilities and allocating time to exercise while at work on the physical activity participation of LEO’s.

Finally, the operational risks associated with law enforcement was the last category identified by our research team during data analysis. Given the relatively small proportion of our participants who identified this category, it appears that the risks associated with police interventions are not a major concern for LEO’s. However, these results might not necessarily imply that LEO’s do not recognize the operational risks of law enforcement. Indeed, it could suggest that LEO’s acknowledge these risks as inherent to law enforcement. Thus, they did not identify the operational risks as an OHSW concern which they feel could be modified.

Some limitations should be acknowledged when interpreting the results from the present study. First, while all LEO’s in Québec were invited to take part in this study, the data analyzed are based on a convenience sample. As a result, female representation in our sample (21.5%) is lower than in the LEO’s population in Québec (27.9%; Gouvernement du Québec, 2021). Also, selection bias may have occurred during the recruiting process. Indeed, it is possible that mainly LEO’s who are particularly unsatisfied with their working conditions provided an answer to our open-ended question. Finally, our results rely exclusively on self-reported answers to an open-ended question. Thus, it is possible that participants may have mainly identified concerns that they consider socially desirable and less stigmatizing.

## Implications for Occupational Health Practice

Given the wide array of occupational hazards associated with law enforcement, the development of OHSW programs tailored to the needs of LEO’s is a challenging task. In the last decades, multiple studies have supported the importance of workers’ engagement throughout the development and implementation of OHSW programs (Feltner et al., 2016; Henning et al., 2009; Strickland et al., 2019). Nevertheless, to our knowledge, the present study is one of the first to provide data on the OHSW concerns of LEO’s. The results from this study provide important information that can help police organizations prioritize future wellness interventions and better target subpopulations that may benefit the most from these interventions. Indeed, while assessing the impact of specific hazards on the OHSW of workers is essential for effective programming, assessing the workers’ perception of their own OHSW is also important as it provides information on their beliefs, attitudes, and knowledge toward these hazards. Overall, our results support that the work schedule along with the occupational stress associated with law enforcement are the most recurrent OHSW concerns of LEO’s. Furthermore, our analyses highlighted some variations in the OHSW concerns of LEO’s based on their level of experience and sex. These results not only stress the need for police organizations to implement OHSW strategies and policies but also suggest that LEO’s are well aware of the negative effects of these hazards and, to some extent, are open to potential interventions or organizational changes that may mitigate the deleterious effects of these hazards. This is important as lack of interest from workers can lead to the failure of OHSW programs.

## References

[bibr1-21650799221134422] Acquadro Maran D. Zedda M. Varetto A . (2018). Physical practice and wellness courses reduce distress and improve wellbeing in police officers. International Journal of Environmental Research and Public Health, 15(4), 578–588. 29570662 10.3390/ijerph15040578 PMC5923620

[bibr2-21650799221134422] Antony J. Brar R. Khan P. A. Ghassemi M. Nincic V. Sharpe J. P. Straus S. E. Tricco A. C. (2020). Interventions for the prevention and management of occupational stress injury in first responders: A rapid overview of reviews. Systematic Reviews, 9(1), 1–20. 32475353 10.1186/s13643-020-01367-w PMC7262749

[bibr3-21650799221134422] Baka L. (2015). The effects of job demands on mental and physical health in the group of police officers. Testing the mediating role of job burnout. Studia Psychologica, 57(4), 285–300.

[bibr4-21650799221134422] Barnes T. D. Beaulieu E. Saxton G. W. (2018). Restoring trust in the police: Why female officers reduce suspicions of corruption. Governance, 31(1), 143–161.

[bibr5-21650799221134422] Benyamina Douma N. Côté C. Lacasse A . (2017). Quebec serve and protect Low back pain study: A web-based cross-sectional investigation of prevalence and functional impact among police officers. Spine, 42(19), 1485–1493. 28248895 10.1097/BRS.0000000000002136

[bibr6-21650799221134422] Brown J. Silvestri M. (2020). A police service in transformation: Implications for women police officers. Police Practice and Research: An International Journal, 21(5), 459–475.

[bibr7-21650799221134422] Carleton R. N. Afifi T. O. Turner S. Taillieu T. El-Gabalawy R. Sareen J. Asmundson G. J. G. (2017). Chronic pain among public safety personnel in Canada. Canadian Journal of Pain, 1(1), 237–246. 35005358 10.1080/24740527.2017.1410431 PMC8730622

[bibr8-21650799221134422] Chen J. C. Chang W. R. Chang W. Christiani D. (2005). Occupational factors associated with low back pain in urban taxi drivers. Occupational Medicine, 55(7), 535–540. 16141293 10.1093/occmed/kqi125

[bibr9-21650799221134422] Cocke C. Dawes J. Orr R. M. (2016). The use of 2 conditioning programs and the fitness characteristics of police academy cadets. Journal of Athletic Training, 51(11), 887–896. 27863188 10.4085/1062-6050-51.8.06 PMC5224730

[bibr10-21650799221134422] Cohen J. (1988). Statistical power analysis for the behavioral sciences. Erlbaum.

[bibr11-21650799221134422] Crompton R. Lyonette C. (2006). Work-life “balance” in Europe. Acta Sociologica, 49(4), 379–393.

[bibr12-21650799221134422] Donnelly C. J. Callaghan J. P. Durkin J. L. (2009). The effect of an active lumbar system on the seating comfort of officers in police fleet vehicles. International Journal of Occupational Safety and Ergonomics, 15(3), 295–307. 19744371 10.1080/10803548.2009.11076809

[bibr13-21650799221134422] Feltner C. Peterson K. Palmieri Weber R. Cluff L. Coker-Schwimmer E. Viswanathan M. Lohr K. N. (2016). The effectiveness of total worker health interventions: A systematic review for a National Institutes of Health pathways to prevention workshop. Annals of Internal Medicine, 165(4), 262–269. 10.7326/M16-0626 27240022

[bibr14-21650799221134422] Garbarino S. Guglielmi O. Puntoni M. Bragazzi N. L. Magnavita N. (2019). Sleep quality among police officers: Implications and insights from a systematic review and meta-analysis of the literature. International Journal of Environmental Research and Public Health, 16(5), 885–900. 30862044 10.3390/ijerph16050885 PMC6427768

[bibr15-21650799221134422] Gendron P. Lajoie C. Laurencelle L. Lemoyne J. Trudeau F. (2020). Physical training in the fire station and firefighters’ cardiovascular health. Occupational Medicine, 70(4), 224–230. 32377669 10.1093/occmed/kqaa060

[bibr16-21650799221134422] Gendron P. Lajoie C. Laurencelle L. Trudeau F. (2019). Cardiovascular health profile among Quebec male and female police officers. Archives of Environmental & Occupational Health, 74(6), 331–340. 29727597 10.1080/19338244.2018.1472063

[bibr17-21650799221134422] Gerber M. Kellmann M. Hartmann T. Pühse U. (2010). Do exercise and fitness buffer against stress among Swiss police and emergency response service officers? Psychology of Sport and Exercise, 11(4), 286–294.

[bibr18-21650799221134422] Gouvernement du Québec. (2021). La desserte policière au Québec: Profil organisationnel 2019. Quebec Ministry of Public Security. https://cdn-contenu.quebec.ca/cdn-contenu/adm/min/securite-publique/publications-adm/publications-secteurs/police/statistiques-desserte-policiere/stats_desserte_policiere_2019.pdf?1641944582

[bibr19-21650799221134422] Green L. W. Kreuter M. W. (2005). Health promotion planning: An educational and ecological approach (4th ed.). McGraw-Hill.

[bibr20-21650799221134422] Gruevski K. M. Holmes M. W. Gooyers C. E. Dickerson C. R. Callaghan J. P. (2016). Lumbar postures, seat interface pressures and discomfort responses to a novel thoracic support for police officers during prolonged simulated driving exposures. Applied Ergonomics, 52(1), 160–168. 26360207 10.1016/j.apergo.2015.07.015

[bibr21-21650799221134422] Henning R. Warren N. Robertson M. Faghri P. Cherniack M. , & CPH-NEW Research Team. (2009). Workplace health protection and promotion through participatory ergonomics: An integrated approach. Public Health Reports, 124(4), 26–35. 10.1177/00333549091244S104 PMC2708654 19618804

[bibr22-21650799221134422] Holmes M. W. R. McKinnon C. D. Dickerson C. R. Callaghan J. P. (2013). The effects of police duty belt and seat design changes on lumbar spine posture, driver contact pressure and discomfort. Ergonomics, 56(1), 126–136. 23140370 10.1080/00140139.2012.739206

[bibr23-21650799221134422] Larsen L. B. Ramstrand N. Tranberg R. (2019). Duty belt or load-bearing vest? Discomfort and pressure distribution for police driving standard fleet vehicles. Applied Ergonomics, 80, 146–151. 31280798 10.1016/j.apergo.2019.05.017

[bibr24-21650799221134422] Lee J. Lee W. R. Yoo K. B. Cho J. Yoon J. (2022). Risk of cerebro-cardiovascular diseases among police officers and firefighters: A nationwide retrospective cohort study. Yonsei Medical Journal, 63(6), 585–590. 35619583 10.3349/ymj.2022.63.6.585 PMC9171666

[bibr25-21650799221134422] Lee M. P. Hudson H. Richards R. Chang C. C. Chosewood L. C. Schill A. L. (2016). Fundamentals of total worker health approaches: Essential elements for advancing worker safety, health, and well-being (DHHS (NIOSH) Publication No. 2017-112). U.S. Department of Health and Human Services, Centers for Disease Control and Prevention, National Institute for Occupational Safety and Health.

[bibr26-21650799221134422] MacEachern A. D. Dennis A. A. Jackson S. Jindal-Snape D. (2019). Secondary traumatic stress: Prevalence and symptomology amongst detective officers investigating child protection cases. Journal of Police and Criminal Psychology, 34(2), 165–174.

[bibr27-21650799221134422] MacMillan F. Karamacoska D. El Masri A. McBride K. A. Steiner G. Z. Cook A. Kolt G. Klupp N. George E. S. (2017). A systematic review of health promotion intervention studies in the police force: Study characteristics, intervention design and impacts on health. Occupational and Environmental Medicine, 74(12), 913–923. 29066612 10.1136/oemed-2017-104430

[bibr28-21650799221134422] Matheson A. O’Brien L. Reid J. A. (2014). The impact of shiftwork on health: A literature review. Journal of Clinical Nursing, 23, 3309–3320. 24460821 10.1111/jocn.12524

[bibr29-21650799221134422] McCreary D. R. Thompson M. M. (2006). Development of two reliable and valid measures of stressors in policing: The operational and organizational police stress questionnaires. International Journal of Stress Management, 13(4), 494–518.

[bibr30-21650799221134422] National Law Enforcement Officers Memorial Fund. (2021). Officer deaths by year. https://nleomf.org/memorial/facts-figures/officer-fatality-data/officer-deaths-by-year/

[bibr31-21650799221134422] Oliver H. Thomas O. Copeland R. J. Hesketh I. Jukes M. Chadd K. Rocca M. (2022). Proof of concept and feasibility of the app-based “# SWPMoveMore challenge”: Impacts on physical activity and well-being in a police population. The Police Journal, 95(1), 170–189.

[bibr32-21650799221134422] Orr R. M. Ford K. Stierli M. (2016). Implementation of an ability-based training program in police force recruits. Journal of Strength and Conditioning Research, 30(10), 2781–2787. 27658234 10.1519/JSC.0000000000000898

[bibr33-21650799221134422] Peterson S. A. Wolkow A. P. Lockley S. W. O’Brien C. S. Qadri S. Sullivan J. P. Czeisler C. A. Rajaratnam S. M. W. Barger L. K. (2019). Associations between shift work characteristics, shift work schedules, sleep and burnout in North American police officers: A cross-sectional study. BMJ Open, 9(11), Article e030302. 10.1136/bmjopen-2019-030302 PMC6924705 31791964

[bibr34-21650799221134422] Queirós C. Passos F. Bártolo A. Marques A. J. Da Silva C. F. Pereira A. (2020). Burnout and stress measurement in police officers: Literature review and a study with the operational police stress questionnaire. Frontiers in Psychology, 11, 587. 32457673 10.3389/fpsyg.2020.00587 PMC7221164

[bibr35-21650799221134422] Rabe-Hemp C. E. (2008). Female officers and the ethic of care: Does officer gender impact police behaviors? Journal of Criminal Justice, 36(5), 426–434.

[bibr36-21650799221134422] Rossomanno C. I. Herrick J. E. Kirk S. M. Kirk E. P. (2012). A 6-month supervised employer-based minimal exercise program for police officers improves fitness. The Journal of Strength and Conditioning Research, 26(9), 2338–2344. 22067246 10.1519/JSC.0b013e31823f2b64

[bibr37-21650799221134422] Rostami H. Tavakoli H. R. Rahimi M. H. Mohammadi M. (2019). Metabolic syndrome prevalence among armed forces personnel (military personnel and police officers): A systematic review and meta-analysis. Military Medicine, 184(9–10), e417–e425. 10.1093/milmed/usz144 31247092

[bibr38-21650799221134422] Saldaña J. (2011). Fundamentals of qualitative research. Oxford University Press.

[bibr39-21650799221134422] Schuck A. M. (2014). Gender differences in policing: Testing hypotheses from the performance and disruption perspectives. Feminist Criminology, 9(2), 160–185.

[bibr40-21650799221134422] Strickland J. R. Kinghorn A. M. Evanoff B. A. Dale A. M. (2019). Implementation of the healthy workplace participatory program in a retail setting: A feasibility study and framework for evaluation. International Journal of Environmental Research and Public Health, 16(4), 590–607. 30781669 10.3390/ijerph16040590 PMC6406806

[bibr41-21650799221134422] Sullivan O. (2019). Gender inequality in work-family balance. Nature Human Behaviour, 3(3), 201–103. 10.1038/s41562-019-0536-3 30953017

[bibr42-21650799221134422] Taylor B. G. Liu W. Mumford E. A. (2021). A national study of the availability of law enforcement agency wellness programming for officers: A latent class analysis. International Journal of Police Science & Management, 24(2), 175–189.

[bibr43-21650799221134422] Tiesman H. M. Gwilliam M. Konda S. Rojek J. Marsh S. (2018). Nonfatal injuries to law enforcement officers: A rise in assaults. American Journal of Preventive Medicine, 54(4), 503–509. 29395571 10.1016/j.amepre.2017.12.005 PMC11323285

[bibr44-21650799221134422] Torquati L. Mielke G. I. Brown W. J. Kolbe-Alexander T. (2018). Shift work and the risk of cardiovascular disease. A systematic review and meta-analysis including dose–response relationship. Scandinavian Journal of Work, Environment & Health, 44(3), 229–238. 10.5271/sjweh.3700 29247501

[bibr45-21650799221134422] Vaismoradi M. Turunen H. Bondas T. (2013). Content analysis and thematic analysis: Implications for conducting a qualitative descriptive study. Nursing & Health Sciences, 15(3), 398–405. 23480423 10.1111/nhs.12048

[bibr46-21650799221134422] Vinstrup J. Jakobsen M. D. Andersen L. L. (2020). Perceived stress and low-back pain among healthcare workers: A multi-center prospective cohort study. Frontiers in Public Health, 8, 297–304. 32850571 10.3389/fpubh.2020.00297 PMC7431956

[bibr47-21650799221134422] Violanti J. M. (2014). Dying for the job: Police work exposure and health. Charles C Thomas Publisher.

[bibr48-21650799221134422] Wolter C. Santa Maria A. Wörfel F. Gusy B. Lesener T. Kleiber D. Renneberg B. (2018). Job demands, job resources, and well-being in police officers: A resource-oriented approach. Journal of Police and Criminal Psychology, 34(1), 45–54.

